# Infection of Human Retinal Pigment Epithelial Cells with Dengue Virus Strains Isolated during Outbreaks in Singapore

**DOI:** 10.3390/microorganisms10020310

**Published:** 2022-01-28

**Authors:** Liam M. Ashander, Amanda L. Lumsden, Abby C. Dawson, Yuefang Ma, Lisia B. Ferreira, Genevieve F. Oliver, Binoy Appukuttan, Jillian M. Carr, Justine R. Smith

**Affiliations:** College of Medicine & Public Health, Flinders University, Adelaide, SA 5042, Australia; liam.ashander@flinders.edu.au (L.M.A.); amanda.lumsden@flinders.edu.au (A.L.L.); abby.dawson@flinders.edu.au (A.C.D.); yuefang.ma@flinders.edu.au (Y.M.); lisia.ferreira@flinders.edu.au (L.B.F.); eyeota@gmail.com (G.F.O.); binoy.appukuttan@flinders.edu.au (B.A.); jill.carr@flinders.edu.au (J.M.C.)

**Keywords:** retina, retinal pigment epithelium, dengue virus, infection, retinopathy, uveitis

## Abstract

Prevalence of dengue retinopathy varies across epidemics, with the disease linked to circulation of dengue virus serotype 1 (DENV-1). The retinal pigment epithelium has been implicated in the pathology. We investigated infectivity, molecular response, and barrier function of epithelial cells inoculated with DENV strains from different outbreaks in Singapore. Monolayers of human retinal pigment epithelial cells (multiple primary cell isolates and the ARPE-19 cell line) were inoculated with six DENV strains, at multiplicity of infection of 10; uninfected and recombinant strain-infected controls were included where relevant. Infectivity and cell response were assessed primarily by RT-qPCR on total cellular RNA, and barrier function was evaluated as electrical resistance across monolayers. Higher viral RNA loads were measured in human retinal pigment epithelial cells infected with DENV-1 strains from the 2005 Singapore epidemic, when retinopathy was prevalent, versus DENV-1 strains from the 2007 Singapore epidemic, when retinopathy was not observed. Type I interferon (IFN) transcripts (IFN-β and multiple IFN-stimulated genes) were up-regulated, and impact on barrier function was more pronounced, for cells infected with DENV-1 strains from the 2005 versus the 2007 Singapore epidemics. Aside from serotype, strain of DENV may determine the potential to induce retinal pathology. Identification of molecular markers of disease-associated DENV strains may provide insights into the pathogenesis of dengue retinopathy.

## 1. Introduction

Eye disease is a recently recognized complication of dengue virus (DENV) infection [1,2]. Reports from Singapore during the 2000s, in particular, have detailed characteristic manifestations that usually involve the posterior segment of the eye [3,4,5,6]. Clinical presentations of dengue retinopathy include diffuse or cystoid macular edema, retinal hemorrhages that may be single or multiple, retinal vasculitis, and ‘orange dot foveolitis’. Good visual acuity is often retained, but a majority of affected individuals experience visual field defects, and foveal dysfunction has been demonstrated by multifocal electroretinogram [7].

Like other flaviviruses, DENV disseminates via circulation, implying it must cross the blood-retinal barrier in order to cause retinopathy [8]. This blood-tissue barrier is based around the retinal endothelium and the retinal pigment epithelium [9]; previous work by our group, using human retinal cell lines and a laboratory-adapted DENV strain, implicated the retinal pigment epithelium in the retinal pathology [10]. Following infection, retinal pigment epithelial cells exhibit a clear cytopathic effect, release DENV in higher titer, and generate a stronger type I interferon (IFN) response than retinal endothelial cells. Furthermore, confluent monolayers of infected retinal pigment epithelial cells show a relatively reduced expression of junctional molecules, which is associated with increased permeability.

Dengue virus exists as four serotypes—DENV-1 through DENV-4 [11]—and ophthalmic surveys have revealed quite different prevalence of eye involvement between outbreaks [12,13]. DENV-1 was the predominant circulating serotype throughout the 2005 epidemic [14], when retinal disease was prevalent [12]: 10% of Singaporean patients infected and hospitalized during this epidemic developed dengue maculopathy. This observation has suggested the possibility that retinal pathology is associated with DENV-1 infections [13]. To investigate this issue, we studied infectivity, molecular responses, and barrier function of multiple primary human retinal pigment epithelial cell isolates, as well as the ARPE-19 human retinal pigment epithelial cell line, following inoculation with DENV strains collected in Singapore across several epidemics, with a focus on DENV-1 strains.

## 2. Materials and Methods

### 2.1. Human Retinal Cells

Human retinal pigment epithelial cells were isolated from human cadaveric eyes (5 male and 3 females, aged 50 to 75 years at death), following a method we have previously published [15]. Death-to-processing time ranged from 15 to 42 h.

Briefly, the retinal pigment epithelium-choroid was digested with 0.25 mg/mL collagenase IA/collagenase IV (Merck-Sigma Aldrich, St Louis, MO, USA) for 30 min at 37 °C and 5% CO_2_ in air. Sheets of retinal pigment epithelium were scraped off the Bruch membrane and subsequently purified by sucrose density gradient centrifugation. The sheets were cultured in 6 cm dishes (growth area = 20 cm^2^) in 50% Minimum Essential Medium Eagle alpha modification (MEM), 25% Dulbecco’s modified Eagle’s medium (DMEM), and 25% F-12 medium, with 1× N1 Medium Supplement, 1× Non-Essential Amino Acids Solution, 1× GlutaMAX Supplement, 0.25 mg/mL taurine, 0.02 μg/mL hydrocortisone, 0.013 ng/mL 3,3′,5-triiodo-L-thyronine sodium, 100 U/mL Penicillin-Streptomycin (all obtained from Merck-Sigma Aldrich or Thermo Fisher Scientific-Gibco, Grand Island, NY, USA), and 2% fetal bovine serum (FBS; Bovogen, Keilor East, Australia), refreshed twice weekly, at 37 °C and 5% CO_2_ in air, and stored frozen in liquid nitrogen on reaching confluence.

The ARPE-19 human retinal pigment epithelial cell line [16] was cultured in DMEM:F12 medium supplemented with 10% FBS (Thermo Fisher Scientific-Gibco), and the human retinal endothelial cell line [17] was cultured in MCDB-131 medium (Merck-Sigma Aldrich) supplemented with 10% FBS (Thermo Fisher Scientific-Gibco) and endothelial growth factors (EGM-2 Single-Quots supplement, omitting gentamicin and hydrocortisone; Clonetics-Lonza, Walkerville, MD, USA), both at 37 °C and in 5% CO_2_ in air.

### 2.2. Dengue Virus Strains

Six DENV field strains were generously provided by Professor Ng Lee Ching, under a Material Transfer Agreement with the Environmental Health Institute of the Singapore National Environment Agency (Agreement EHI-132), representing DENV-1 and DENV-2 isolated in Singapore between 2004 and 2007 (Table 1). These isolates were propagated in C6/36 mosquito cells (American Type Culture Collection, Gaithersburg, MA, USA) in Eagle’s basal medium, 1 mM sodium pyruvate, 2 mM L-glutamine, 10 mM HEPES, and 7.5% FBS (all from Thermo Fisher Scientific-Gibco). A seventh strain, the full-length, infectious recombinant DENV-2, Mon601, was originally cloned from a mouse brain-adapted New Guinea C isolate [18], and viral RNA was transfected into baby hamster kidney BKH-21 fibroblasts before amplification in C6/36 mosquito cells [19].

The DENV field strains were titered against the Mon601 DENV strain by quantitative real-time polymerase chain reaction (qPCR), using RNA extracted from culture supernatant with the High Pure Viral Nucleic Acid Kit (Roche Life Sciences, Mannheim, Germany). The Mon601 strain was separately titered by neutral red plaque assay on Vero cells, as previously described [20], and expressed as plaque forming units (pfu)/mL. This permitted an equivalent viral genome/pfu to be calculated across the seven DENV strains.

### 2.3. Infection of Retinal Cells with Dengue Virus

After seeding in 12-well plates (growth area = 4 cm^2^) for RNA isolation, in 96-well plates (growth area = 0.3 cm^2^) for immunolabelling, and in E-plate L8 tissue culture plates (ACEA Biosciences-Agilent, Hangzhou, China: growth area = 64 mm^2^), confluent cell monolayers were infected with individual DENV strains at a multiplicity of infection (MOI) of 10, in a minimal volume of DMEM containing no additives. Control cell monolayers were exposed to the same volume of fresh DMEM alone. Following a 90-minute incubation at 37 °C and in 5% CO_2_ in air, and rocking the dishes every 15 min, cell-specific culture medium was added in standard volumes, and the infected and uninfected cell monolayers were returned to incubation for pre-determined time intervals. Primary cells were used at passage 2 or less.

### 2.4. RNA Extraction and Reverse Transcription

Extraction of total RNA from infected and uninfected cells was performed using Trizol Reagent (Thermo Fisher Scientific-Ambion, Carlsbad, CA, USA), according to the manufacturer’s instructions. The concentration of recovered nucleic acid was determined using the Nanodrop 2000 spectrophotometer (Thermo Fisher Scientific, Wilmington, DE, USA). Reverse transcription (RT) was carried out using iScript Reverse Transcription Supermix for RT-qPCR (Bio-Rad, Hercules, CA, USA). Total RNA input was 200 ng per reaction, yielding 20 uL of cDNA. Two reactions were prepared for each sample, and the resulting cDNA was pooled and used at a dilution of up to 1:10 in qPCR.

### 2.5. Dengue Virus Primers

To design a primer set that would detect all DENV strains, we extracted and aligned cDNA sequences of the six field isolates and Mon601, using the GenBank genetic sequence database [21] and the Clustal Omega multiple sequence alignment program [22]. Complete cDNA sequences were available for the DENV-2 strains (Mon601 [AF038403.1], EHI0377Y04 [JN851123.1], and EHI0578Y05 [JN851126.1]), whereas partial sequences in the envelope protein region were available for the DENV-1 strains (EHI0393Y04 [EU069606.1], EHI0418Y05 [EU069594.1], EHI00043Y07 [GQ357691.1], and EHI0169Y07 [GQ357690.1]).

Although stretches of complete homology were evident within the envelope region, none were sufficiently long to design primer pairs with no mismatches of base pairs (bp) across all DENV strains. Optimal primer pairs were designed using a multiple alignment-based primer design algorithm [23], with user-defined criteria of amplicon size ≤ 400 bp and degenerate bp ≤ 3. Sets that were not conserved within the final 3 bp at the 3′ end were rejected, and for the remaining options, bp that were not matched across all sequences were replaced with bp that mismatched for all sequences, so as not to favor one DENV strain over another. Finally, sets with complementarity with the last 3 bp and sets with the potential to detect targets in the human mRNA RefSeq NCBI Reference Sequence Database [24] were excluded.

The DENV primer pair, designed as described, had two mismatches in the forward primer (positions 15 and 16 of 23 nucleotides) and two mismatches in the reverse primer (positions 6 and 12 of 20 nucleotides) and generated an amplicon of 200 bp across all DENV strains. Amplicons were confirmed by sequencing. This primer pair, along with all other primer pairs used in this work, are presented in Table 2.

### 2.6. Polymerase Chain Reaction

Quantitative real-time PCR was performed using the CFX Connect Real-Time PCR Detection System (Bio-Rad), with either iQ SYBR Green Supermix or SsoAdvanced Universal SYBR Green Supermix (both obtained from Bio-Rad), and primers (synthesized by Merck-Sigma Aldrich) used at a concentration of 0.3 uM. The standard amplification consisted of: a pre-cycling hold at 95 °C for 5 min; 40 cycles of denaturation for 30 s at 95 °C; annealing for 30 s at 59 °C; extension for 30 s at 72 °C; and a post-extension hold at 75 °C for 1 s. A melting curve was analyzed to confirm the purity of each amplicon, and the quantification cycle was measured using the single threshold method. Relative viral load and host gene expression were determined by the Pfaffl method [32], and normalized to stable reference genes, TATA box-binding protein (TBP), or ribosomal protein lateral stalk subunit P0 (RPLP0). Efficiencies were 63–76% for the DENV primer set across the different strain sequences, and greater than 85% for all other primer sets.

### 2.7. Cellular Immunolabelling

Infected and uninfected cell monolayers were fixed with 4% paraformaldehyde for up to 15 min at room temperature, and held in phosphate buffered saline (PBS) at 4 °C prior to labelling for double-stranded RNA with the J2 anti-double-stranded RNA mouse monoclonal antibody (English and Scientific Consulting, Szirak, Hungary). The monolayers were blocked with PBS containing 4% bovine serum albumin (Sigma Aldrich) and 4% normal goat serum (Vector Labs, Burlingame, CA, USA: catalogue number = 10010500) at room temperature, incubated overnight at 4 °C with J2 or isotype-matched (mouse immunoglobulin [IgG]2a_κ_) negative control antibody (BD Pharmingen, San Jose, CA, USA: catalogue number = 550339), diluted to 5 ug/mL in blocking solution, and subsequently labelled with AlexaFluor 488-tagged goat anti-mouse IgG (Thermo Fisher Scientific-Molecular Probes, Eugene, OR, USA: catalogue number = A11029) for 45 min at room temperature. Nuclei were stained with 4′,6 diamidino-2-phenylindole (Merck-Sigma Aldrich) prior to application of Fluoromount Aqueous Mounting Medium (Merck-Sigma Aldrich). The labelled monolayers were photographed under fluorescence at the IX53 inverted microscope (Olympus Corporation, Tokyo, Japan).

### 2.8. Measurement of Transcellular Electrical Resistance

Transcellular electrical resistance was measured in real time with the RTCA iCELLigence (ACEA Biosciences-Agilent, San Diego, CA, USA), and expressed as ‘cell index’, a unitless measure that is calculated by comparison of seeded and empty wells [33]. After seeding in the E-plates, cell monolayers were rested for 30 min at 37 °C and in 5% CO_2_ in air, and subsequently placed in the RTCA iCELLigence. At 48 h, E-plates were removed from the machine, and DENV strains were added to the cell monolayers. After the 90-minute infection interval, the E-plates were returned to the RTCA iCELLigence. Measurements of transcellular electrical resistance were taken every hour for the subsequent 4 days. The cell indices of infected and uninfected monolayers in the same E-plate were required to be equal (*p*-value greater than 0.05) immediately prior to infection, to ensure that any differences observed between conditions were due to infection with DENV, and not cell-related factors.

### 2.9. Statistical Analyses

Data were analyzed using GraphPad Prism (GraphPad Software, La Jolla, CA, USA). The Kruskal–Wallis test and the Mann–Whitney U test were used to compare viral load and host gene expression across the viral strains. Cell indices were compared between infected and uninfected individual cell isolates by unpaired Student’s *t*-test. Extreme deviations in the cell index may occur due to factors such as faulty electrical contact or uneven cell seeding; a well was excluded from analysis if the cell index at 24 h after infection fell three standard deviations outside the other measurements, as recommended by others [34]. For all testing, a statistically significant difference was defined by a *p*-value of less than 0.05.

### 2.10. Human Research Compliance

Use of human cadaver donor eyes from the Eye Bank of South Australia (Adelaide, Australia) for this work was approved by the Southern Adelaide Clinical Human Research Ethics Committee (protocol number: 175.13).

## 3. Results

### 3.1. Human Retinal Pigment Epithelial Cells and Retinal Vascular Endothelial Cells Are Differentially Susceptible to Infection with Dengue Virus Field Strains

**Figure 1:**
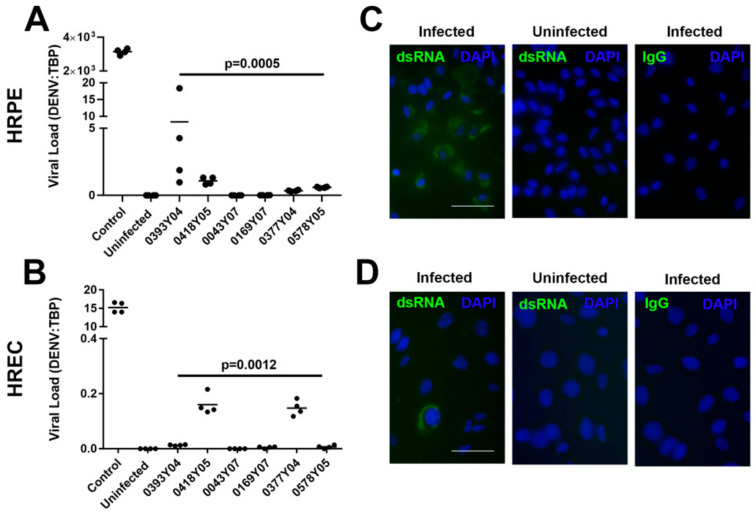
Susceptibility of human retinal pigment epithelial cells and vascular endothelial cells to infection with DENV-1 and DENV-2 strains: DENV-1 viral strains = Singapore National Environmental Agency Isolate (EHI)0393Y04, EHI0418Y05 (from 2005 epidemic), EHI0043Y07 and EHI0169Y07 (from 2007 epidemic); DENV-2 viral strains = EHI0377Y04 and EHI0578Y05 (from 2005 epidemic); HRPE = ARPE-19 human retinal pigment epithelial cell line; HREC = human retinal vascular endothelial cell line, produced in-house; multiplicity of infection = 10; inoculation interval = 90 min; time of infection = 48 h. (**A**,**B**) Graphs showing viral load, expressed as DENV transcript relative to TATA box-binding protein (TBP). Dots represent individual cell monolayers (*n* = 4 monolayers per condition) and crossbars represent means. Control was the laboratory-adapted Mon601 strain. Data were analyzed by Kruskal–Wallis test. (**C**,**D**) Representative photomicrographs of cell monolayers infected with Mon601 and immunolabelled for dsRNA or negative control immunoglobulin (IgG). Alexa Fluor 488 (green) with 40,6-diamidino-2-phenylindole (DAPI) nuclear counterstain (blue). Scale bar measures 50 microns.

To investigate the possibility that the DENV-1 serotype was associated with retinal pathology, we examined interactions between human retinal pigment epithelial cells and a range of DENV strains isolated in Singapore during recent dengue epidemics. We studied two DENV-1 strains isolated immediately before and during the 2005 epidemic (when DENV-1 was the predominant circulating serotype, and retinal involvement was prevalent [12]), two DENV-1 strains isolated during the 2007 epidemic (when DENV-1 was not the predominant circulating serotype and retinal involvement was not reported [13]), and two DENV-2 strains isolated immediately before and during the 2005 epidemic. We first confirmed the relative infectivity of the pigment epithelial cells to infection with the DENV field strains, in comparison to vascular endothelial cells. Monolayers of ARPE-19 human retinal pigment epithelial cells and a characterized human retinal vascular endothelial cell line were inoculated with one of the six DENV field strains at MOI of 10. In parallel, additional monolayers were infected with the Mon601 laboratory-adapted DENV-2 strain as a positive control, or left uninfected. At 48 h following the infection, viral load was quantified by RT-qPCR of total RNA extracted from the cell monolayers, and fixed cells were immunolabelled for viral RNA (Figure 1).

As anticipated, infectivity of all field isolates was lower than the laboratory-adapted strain. Overall, viral RNA load was considerably higher in the pigment epithelial cells in comparison to the vascular endothelial cells. However, for both cell populations, there was a significant difference in viral loads across the field isolates (Figure 1A,B (*p* ≤ 0.0012)): higher viral loads were measured for DENV-1 strains from the 2005 epidemic (EHI0393Y04 and EHI0418Y05) than for DENV-1 strains from the 2007 epidemic (EHI0043Y07 and EHI0169Y07), which were barely detected. Viral loads measured for DENV-2 strains from the 2005 epidemic (EHI0377Y04 and EHI0578Y05) were also higher than DENV-1 strains from the 2007 epidemic. Consistent with its relatively higher infectivity, only Mon601 was detectable by immunolabelling of inoculated cells, with more pigment epithelial cells infected than vascular endothelial cells (Figure 1C,D). Taken together, these data show that human retinal pigment epithelial cells are susceptible to infection with DENV field isolates, with higher infectivity than retinal vascular endothelial cells. Thus, subsequent experiments focused on human retinal pigment epithelial cells. Notably, in these experiments, DENV strain, rather than serotype, appeared to determine infectivity.

### 3.2. Human Retinal Pigment Epithelial Cells Respond Differently to Infection with Different Dengue Virus Field Strains

**Figure 2:**
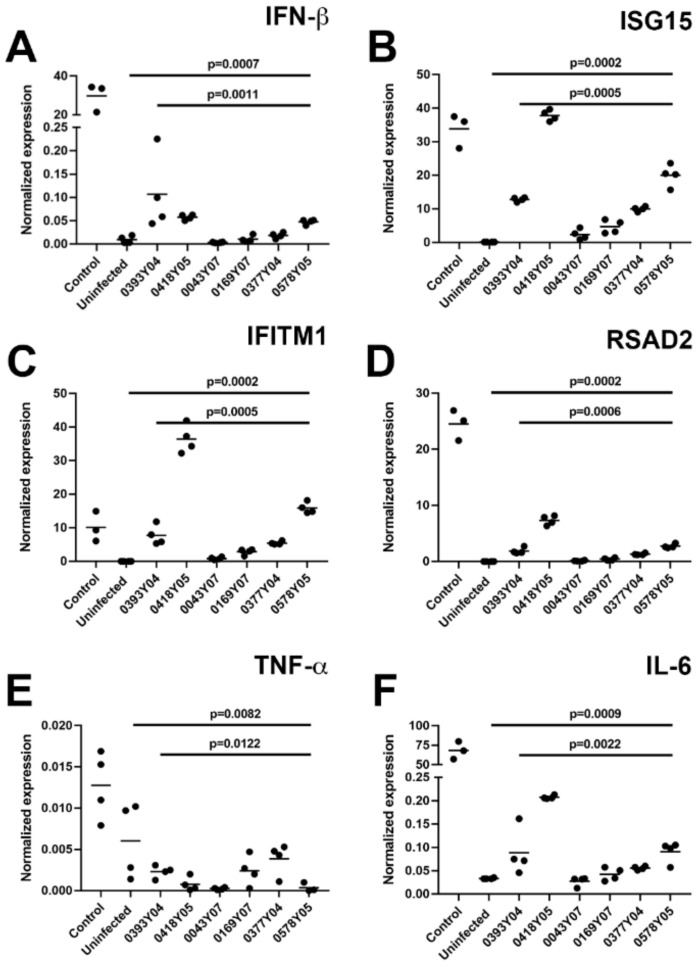
Molecular response of human retinal pigment epithelial cells to infection with DENV-1 and DENV-2 strains: DENV-1 viral strains = Singapore National Environmental Agency Isolate (EHI)0393Y04, EHI0418Y05 (from 2005 epidemic), EHI0043Y07 and EHI0169Y07 (from 2007 epidemic); DENV-2 viral strains = EHI0377Y04 and EHI0578Y05 (from during 2005 epidemic); ARPE-19 human retinal pigment epithelial cell line; multiplicity of infection = 10; inoculation interval = 90 min; time of infection = 48 h. (**A**–**F**) Graphs showing cellular expression of transcript relative to TATA box-binding protein (TBP): IFN-β = interferon-β, IFITM1 = IFN-induced transmembrane protein 1, RSAD2 = radical SAM domain-containing 2, ISG15 = IFN-stimulated gene 15, TNF-α = tumor necrosis factor-α, IL-6 = interleukin-6. Dots represent individual cell monolayers (*n* = 3–4 monolayers per condition), and crossbars represent means. Control was the laboratory-adapted Mon601 strain. Data were analyzed by Kruskal–Wallis test.

The anti-viral and inflammatory responses of human retinal pigment epithelial cells to infection with different DENV strains were investigated in ARPE-19 human retinal pigment epithelial cell monolayers 48 h after inoculation by RT-qPCR of representative transcripts (Figure 2). The anti-viral type I interferon (IFN) response is dominated by IFN-β, and the many IFN-stimulated genes that are released in response to its production, including IFN-induced transmembrane protein 1 (IFITM1), radical SAM domain-containing 2 (RSAD2, also known as viperin), and IFN-stimulated gene 15 (ISG15) [35]. There was a significant difference in the levels of IFN-β, IFITM1, RSAD2, and ISG15 transcripts produced in response to the different DENV field strains (*p* ≤ 0.001), with relatively high levels induced by DENV-1 strains from the 2005 epidemic (EHI0393Y04 and EHI0418Y05) and relatively low levels induced by DENV-1 strains from the 2007 epidemic (EHI0043Y07 and EHI0169Y07); DENV-2 strains from the 2005 epidemic (EHI0377Y04 and EHI0578Y05) also induced more transcript than DENV-1 strains from the 2007 epidemic (Figure 2A–D). Uninfected control monolayers produced little IFN-β and negligible detectable ISGs, and monolayers infected with Mon601 laboratory-adapted DENV-2 produced high levels of these transcripts. Tumor necrosis factor (TNF)-α and interleukin (IL)-6 are major inflammatory cytokines produced by retinal pigment epithelial cells. Induction and significant differences in IL-6 transcript expression were seen across the strains (*p* = 0.002), with higher levels induced by the 2005 epidemic DENV-1 and DENV-2 strains in comparison to the 2007 epidemic DENV-1 strains (Figure 2F). Although significant differences also were seen for TNF-α transcript, in contrast to IL-6, there was low induction overall, even by Mon601 (Figure 2E). Overall, these observations are consistent with the viral infectivity results, with strain rather than serotype determining the human retinal pigment epithelial cell response to DENV infection.

### 3.3. Viral Load and Cell Response Are Different in Human Primary Retinal Pigment Epithelial Cells Infected with Different Dengue Virus Serotype 1 Field Strains

**Figure 3:**
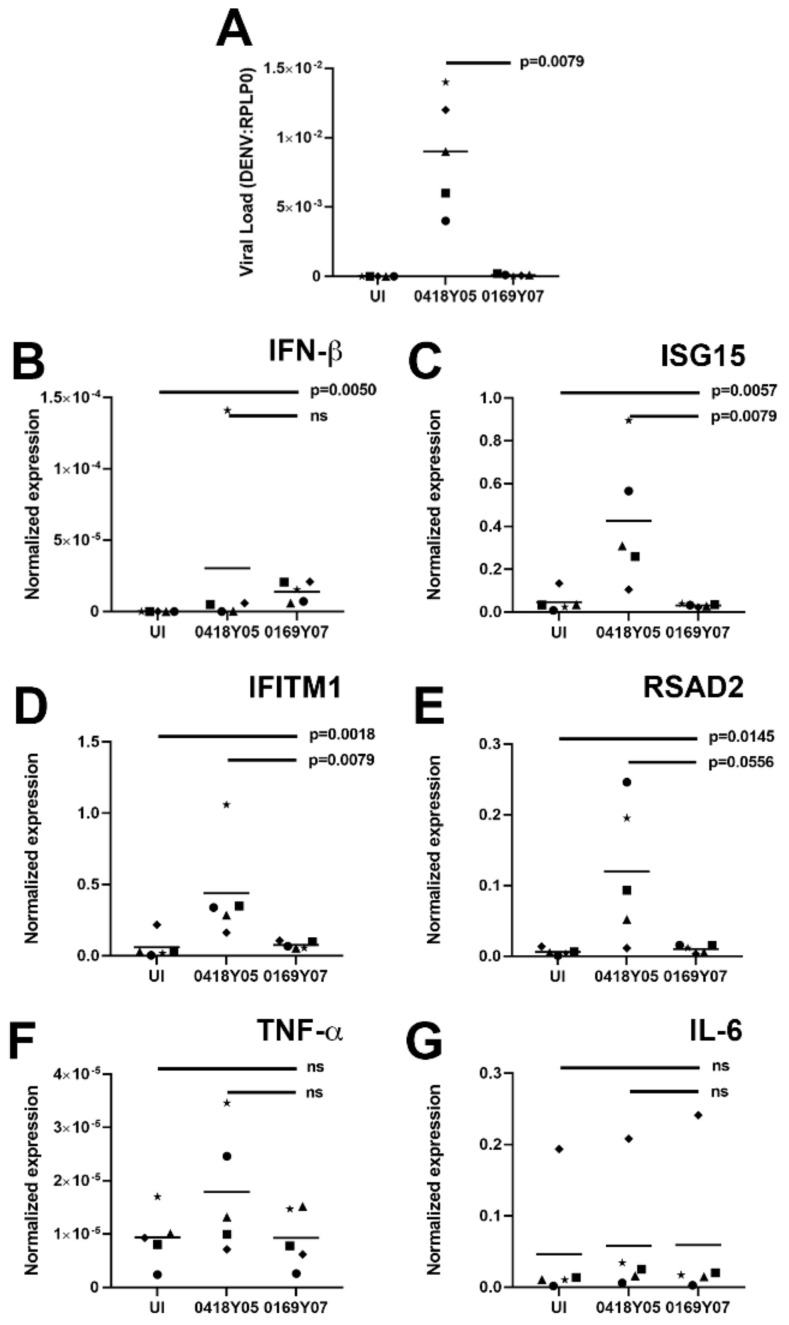
Interaction of primary human retinal pigment epithelial cells with DENV-1 strains: DENV-1 viral strains = Singapore National Environmental Agency Isolate (EHI)0418Y05 (isolated during 2005 epidemic) and EHI0169Y07 (isolated during 2007 epidemic); *n* = 5 primary human retinal pigment epithelial cell isolates; multiplicity of infection = 10; inoculation interval = 90 min; time of infection = 48 h. (**A**) Graph showing viral load expressed as DENV transcript relative to ribosomal protein lateral stalk subunit P0 (RPLP0). (**B**–**G**) Graphs showing cellular expression of transcript relative to RPLP0: IFN-β = interferon-β, IFITM1 = IFN-induced transmembrane protein 1, RSAD2 = radical SAM domain-containing 2, ISG15 = IFN-stimulated gene 15, TNF-α = tumor necrosis factor-α, IL-6 = interleukin-6. Shapes represent individual cell isolates, and crossbars represent means. Statistical comparisons were made across three groups by Kruskal–Wallis test and across two groups by Mann–Whitney U test.

The ARPE-19 human retinal epithelial cell line exhibits differences from primary human cells, including in the setting of infection [30]. Also, there are differences in gene expression between individuals that may be considerable [30,36,37]. Therefore, we sought to confirm the key finding of a difference in infectivity and host response between DENV-1 strains from the 2005 versus the 2007 Singapore epidemics using retinal pigment epithelial cells individually isolated from eyes of five different human donors. Cells from five donors were inoculated with EHI0418Y05 (isolated during the 2005 epidemic) or EHI0169Y07 (isolated during the 2007 epidemic) at MOI of 10, and viral load and host response were studied by RT-qPCR on RNA extracted 48 h later. These experiments demonstrated that infection with EHI0418Y05 resulted in a significantly higher viral RNA load than EHI0169Y07 (*p* = 0.0079) (Figure 3A). Infections with both EHI0418Y05 and EHI0169Y07 elicited a significant rise in IFN-β transcript (*p* ≤ 0.001) (Figure 3B), but IFN-stimulated genes, IFITM1, RSAD2, and ISG15, were expressed at higher levels following infection with EHI0418Y05 than with EHI0169Y07 (Figure 3C–E). However, there was no significant increase in expression of inflammatory cytokines, TNF-α and IL-6, over the baseline level that was measured in uninfected cells (*p* > 0.05) (Figure 3F,G). These results confirm the susceptibility of human retinal pigment epithelial cells to infection with DENV field isolates, and support a difference between the infectivity of, and Type I IFN response to, DENV-1 strains from the 2005 and the 2007 Singapore epidemics.

### 3.4. Dengue Virus Serotype 1 Field Strains Have Differential Effects on Permeability of Human Primary Retinal Epithelial Cell Monolayers

**Figure 4:**
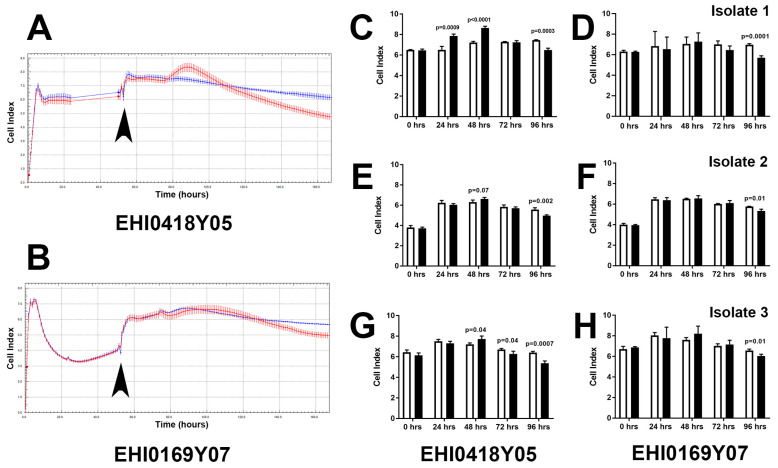
Effect of DENV-1 infection on barrier function of primary human retinal pigment epithelial cells: viral strains = Singapore National Environmental Agency Isolate (EHI)0418Y05 (isolated during 2005 epidemic) and EHI0169Y07 (isolated during 2007 epidemic); *n* = 3 primary human retinal pigment epithelial cell isolates, studied and presented separately; multiplicity of infection = 10; inoculation interval = 90 min; time of infection = 4 days. (**A**,**B**) Representative plots of transcellular electrical resistance across (**A**) EHI0418Y05-infected or (**B**) EHI0169Y07-infected (red) versus uninfected (blue) cell monolayers, measured as cell index each hour. Arrowheads mark time of return to measurements following inoculation. Dots represent mean, with error bars indicating standard deviation; *n* = 3–4 monolayers per condition. (**C**–**H**) Graphs showing cell index at specified time intervals following inoculation for (**C**,**E**,**G**) EHI0418Y05-infected or (**D**,**F**,**H**) EHI0169Y07-infected (black) versus uninfected (white) cell monolayers for three individual primary cell isolates. Bars represent mean, with error bars showing standard deviation; *n* = 3–4 monolayers per condition. Data were analyzed by 2-tailed Student’s *t*-test.

The retinal pigment epithelium is a key component of the outer blood-retinal barrier [9]. We used the RTCA iCELLigence biosensor to evaluate changes in transcellular electrical resistance of primary human retinal pigment epithelial cell monolayers infected with EHI0418Y05 and EHI0169Y07 DENV-1 strains from the 2005 and 2007 Singapore epidemics, respectively (Figure 4A,B). Similar results were obtained for isolates prepared from three different human donors. Overall changes in barrier function were more marked following infection with EHI0418Y05: in comparison to uninfected cells, a significant increase in resistance occurred 24–48 h post-infection, and a significant decrease in resistance occurred at 72–96 h post-infection (Figure 4C,E,G). For infection with EHI0169Y07, there was no early change in resistance in comparison to uninfected cells, but a significant decrease in resistance was observed at 96 h (Figure 4D,F,H). These data demonstrate the potential for DENV-1 infection to alter barrier function of the human retinal pigment epithelium, and show a difference in the effect of DENV-1 strains isolated during the 2005 and 2007 Singapore epidemics.

## 4. Discussion

Rigorous clinical studies from Singapore have demonstrated substantial variation in the prevalence of retinopathy during dengue epidemics when different DENV serotypes predominated [12,13]. One logical explanation for this phenomenon is that different DENV serotypes are differentially pathogenic in the retina. Clinical observations and laboratory studies have implicated the retinal pigment epithelium in dengue retinopathy [1,10]. We found significantly higher viral loads in human retinal pigment epithelial cells infected with DENV-1 from the 2005 epidemic, when retinopathy was prevalent, versus DENV-1 from the 2007 epidemic, when retinopathy was not observed. Moreover, the retinal pigment epithelial cell type I IFN response was more robust, and the impact on cellular barrier function was more pronounced, following infection with strains from the 2005 epidemic compared to those from the 2007 epidemic. Our findings suggest that DENV strain-related differences may impact the potential for retinal disease.

In addition to multiple roles in retinal homeostasis, including maintenance of the blood-retinal barrier, the retinal pigment epithelium has been identified as a key host cell for a range of micro-organisms, including some bacteria and parasites (e.g., *Mycobacteria tuberculosis* and *Toxoplasma gondii*) [38,39], and a host of different viruses including flaviviruses (e.g., DENV, Zika virus [ZIKV], West Nile virus), herpesviruses (e.g., herpes simplex virus, varicella zoster virus, cytomegalovirus), and filoviruses (i.e., Zaire ebolavirus) [40,41,42,43,44,45]. Ophthalmic imaging studies in patients have documented changes implicating the retinal pigment epithelium in other infectious diseases (e.g., syphilis and coronavirus disease 2019) [46,47]. Two prior studies have explored the susceptibility of the human retinal pigment epithelium to infection with DENV, including our previous report [10] and the report by Singh and associates that focused on ZIKV infection [40]; however, both these studies used DENV-2 strains, rather than DENV-1 strains.

Dengue retinopathy is characterised by various inflammatory lesions and complications, including foveolitis, retinal vasculitis, and macular edema [1]. The inflammatory cytokines, TNF-α and IL-6, are strongly implicated in the development of non-infectious retinal inflammation and the macular oedema that often accompanies this [48,49]. However, we observed no induction of TNF-α following infection of ARPE-19 or primary retinal pigment epithelial cells with DENV-1 strains, and induction of IL-6 in infected ARPE-19 cells alone. On the contrary, there was a significant upregulation of the type I IFN response in both ARPE-19 cells and primary retinal pigment epithelial cells, as indicated by increased expression of ISG15, IFITM1, and RSAD2, and, in the case of ARPE-19 cells, IFN-β. Importantly, this response was significantly higher in primary cell isolates infected with a DENV-1 strain from the 2005 Singapore epidemic than the same isolates infected with a DENV-1 strain from the 2007 epidemic. Although recognized as the major mechanism for controlling viral infection, a chronic or dysregulated type I IFN response has been linked to a range of systemic autoimmune diseases [50]; recently Roy and co-workers demonstrated involvement of this pathway in neuroinflammation in both animal models and human pathological specimens [51]. Thus, a type I IFN response may drive inflammatory pathology in dengue retinopathy, and our results are consistent with more pathology induced by DENV-1 strains from the 2005 Singapore epidemic in comparison to the 2007 epidemic.

Although the predominant DENV serotype circulating in the 2005 epidemic in Singapore was DENV-1, all viral serotypes are endemic and circulate in Singapore [52], and patients with maculopathy have not been tested for DENV serotype [12]. Indeed, severe dengue has been more closely associated with DENV-2 compared to other serotypes [53,54]. Nonetheless, our results support the potential for DENV-1 to induce eye disease, including viral replication and a host cell response that may augment pathology in the eye. The impact of DENV serotype on tissue barriers presented by different vascular endothelia—including the retinal endothelium—was explored in a study by Soe and co-workers [55], using single examples of the four DENV serotypes obtained from different geographic locations. Interestingly, in experiments using a biosensor to follow transcellular electrical resistance, comparable to our experiments, DENV-1 induced the most marked changes in resistance compared to other DENV serotypes, with an initial decrease and subsequent increase in monolayer permeability. We observed a similar phenomenon in the 2005, but not the 2007, DENV-1 field strain. We did not study DENV-3 or DENV-4 strains as our investigation was directed clinically, towards DENV-1; future research around viral biology could evaluate interactions between human retinal pigment epithelial cells and a spectrum of strains across all DENV serotypes.

Our investigation made use of six DENV field strains (Table 1). Clinical manifestations associated with infection by these specific DENV strains have not been reported. In fact, although multiple studies have correlated DENV serotype with clinical parameters [56,57,58,59], there has been little work on DENV strain and genotype, and disease features. Suppiah and colleagues observed that different DENV-3 strains were differentially associated with musculoskeletal symptoms in patients suffering from dengue without warning signs [60]. Zhang and co-workers demonstrated differences in the amount of weight lost by C57BL/6 IFN-α/β receptor knockout mice infected with different DENV-2 strains [61]. Like these findings, our results suggest that there is value in considering the infectivity and biological effects of individual strains, regardless of serotype, when studying the pathogenic mechanisms of DENV infection.

Our work has focused on retinal pigment epithelial cells specifically, consistent with their role in the blood-retinal barrier and ocular inflammation, and their relative susceptibility to DENV infection. However, other retinal cell populations, including glial cells and neuronal subsets, also may participate in the pathogenesis of retinopathy. Given that we were studying disease mechanisms in a human system, our work was conducted in vitro, and potential limitations of the cell culture system must be acknowledged. The ARPE-19 cell is a common model for human retinal pigment epithelial cells, being readily obtainable and easy to culture [16]. However, the transcriptome of the ARPE-19 cell differs from that of primary human retinal pigment epithelial cells [62], and we have observed differences in molecular responses of ARPE-19 cells and primary cells in the setting of infection (i.e., cell responses to infection with *T. gondii*) [63,64]. For this reason, we confirmed our observations made in ARPE-19 cells in early passage primary human retinal pigment epithelial cells. Given the potential for interindividual differences in cell susceptibility and response to viral infection, we worked with multiple human donor isolates that were prepared independently.

In conclusion, the research findings we have presented here raise the possibility that DENV strains, rather than or in addition to DENV serotypes, have different potential to result in retinal pathology. Future studies could be directed at exploring this conclusion using DENV strains isolated from outbreaks in other regions and/or at other times. In addition, the identification of molecular markers of disease-associated DENV strains may provide insights into the pathogenesis of dengue retinopathy.

## Figures and Tables

**Table 1:** Dengue virus field strains.

Name	Year Isolated	Serotype	GenBank ID
EHI0393Y04	2004	DENV-1	EU069606.1
EHI0418Y05	2005	DENV-1	EU069594.1
EHI0377Y04	2004	DENV-2	JN851123.1
EHI0578Y05	2005	DENV-2	JN851126.1
EHI0043Y07	2007	DENV-1	GQ357691.1
EHI0169Y07	2007	DENV-1	GQ357690.1

**Table 2:** Primer pairs and product sizes for gene transcripts.

Transcript [Reference]	Primer Pair *	Product Size (bp)
DENV	Forward 5′-AAACCAACATTGGAgcTTGAACT-3′ Reverse 5′-CCATTcCCCCAaCCTCTGTC-3′	200
IFITM1 [25]	Forward 5′-ACTCCGTGAAGTCTAGGGACA-3′ Reverse 5′-TGTCACAGAGCCGAATACCAG-3′	155
IFNB [26]	Forward 5′-AAACTCATGAGCAGTCTGCA-3′ Reverse 5′-AGGAGATCTTCAGTTTCGGAGG-3′	168
IL-6 [27]	Forward 5′-ATGAACTCCTTCTCCACAAGCGC-3′ Reverse 5′-GAAGAGCCCTCAGGCTGGACTG-3′	628
ISG15 [28]	Forward 5′-GAGAGGCAGCGAACTCATCT-3′ Reverse 5′-AGCATCTTCACCGTCAGGTC-3′	99
RSAD2 [29]	Forward 5′-TGACGGAACAGATCAAAGCA-3′ Reverse 5′-GCACCAAGCAGGACACTTCT-3′	174
RPLP0 [30]	Forward 5′-GCAGCATCTACAACCCTGAA-3′ Reverse 5′-GCAGATGGATCAGCCAAGAA-3′	235
TBP [31]	Forward 5′-GCCTCCCCCACCCCCTTCTTT-3′ Reverse 5′-GCCACACCCTGCAACTCAACATCC-3′	106
TNFA [27]	Forward 5′-TCTCGAACCCCGAGTGACAA-3′ Reverse 5′-TGAAGAGGACCTGGGAGTAG-3′	181

Abbreviations: bp = base pairs; DENV = dengue virus; IFITM1 = interferon induced transmembrane protein 1; IFNB = interferon-β; IL-6 = interleukin-6; ISG15 = interferon-stimulated gene 15 ubiquitin-like modifier; RSAD2 = radical S-adenosyl methionine domain containing 2 (also known as viperin); RPLP0 = ribosomal protein lateral stalk subunit P0; TBP = TATA-box binding protein; TNFA = tumor necrosis factor-α. * Lower case letters indicate bp that mismatch across all six DENV field strains and the laboratory-adapted Mon601 strain.

## Data Availability

Data sharing is not applicable to this article. The data described in this article are presented in full in the article.
